# Multi-omics reveals the associations between cecal candidate bacteria and muscle fatty acid composition variations in Daheng and Luning chickens

**DOI:** 10.3389/fmicb.2026.1907226

**Published:** 2026-08-31

**Authors:** Daozi Zhaxi, Chunlin Yu, Jiayan Wang, Dongmei Yang, Zi Wang, Wenqi Yang, Yuding Zhang, Chaowu Yang, Zhixiong Li

**Affiliations:** 1 Key Laboratory of Qinghai-Tibetan Plateau Animal Genetic Resource Reservation and Utilization of Ministry of Education, Southwest Minzu University, Chengdu, China; 2 Key Laboratory of Animal Science of National Ethnic Affairs Commission of China, Southwest Minzu University, Chengdu, China; 3 College of Animal & Veterinary Sciences, Southwest Minzu University, Chengdu, China; 4 Sichuan Animal Science Academy, Chengdu, China

**Keywords:** chicken, fatty acid, gut-muscle axis, meat quality, multi-omics

## Abstract

**Introduction:**

Accumulating evidence underscores the profound impact of the gastrointestinal ecosystem on skeletal muscle lipid dynamics.

**Methods:**

This study was designed to investigate the systemic associations connecting the gut microbiota, intermediate metabolomes, and intramuscular fatty acid (FA) deposition in chicken.

**Results:**

Phenotypic and lipophilic profiling revealed distinct FA compositions between Daheng chickens (DHC) and Luning chickens (LNC), with DHC exhibiting significantly higher concentrations of 26 distinct FAs including flavor-precursor MUFAs (C18:1n9c) and functional omega-3 (n-3) PUFAs (C20:5n3 and C22:5n3) within the leg muscle. High-throughput metagenomic sequencing subsequently identified positive correlations between dominant cecal bacterial taxa and these specific muscle FAs. Concurrently, multi-compartment metabolomics mapped a spectrum of differential metabolites spanning the cecum, serum, and leg muscle, which were fundamentally enriched in the “cysteine and methionine metabolism” and “pantothenate and CoA biosynthesis” pathways. Through an integrative multi-omics framework paired with statistical mediation modeling, we identified a candidate functional axis: the *Agathobaculum*-*Collinsella*-*Enorma* consortium strongly co-varies with muscle FA deposition, a process bridged by key intermediate nodes including Pyruvic acid, S-adenosylmethionine, and Spermine.

**Conclusion:**

Collectively, these findings suggest that the gut microbiota is closely coupled with muscle lipid composition via a structured gut-metabolite-muscle axis, offering candidate microbial markers for precision management of meat quality in poultry production.

## 1 Introduction

Chickens are among the most widely distributed and economically important livestock species worldwide, with strong adaptability to diverse climates and regions. The reliance on chickens as a primary protein source has resulted in their burgeoning numbers across continents. According to statistics from the Food and Agriculture Organization (FAO), the global chicken population has risen consistently and substantially over the past six decades, increasing from nearly 4 billion in 1961 to more than 34 billion in 2023. As the second most consumed meat worldwide by humans, chicken is favored for its deliciousness and juiciness, which has further raised industry and consumer demands for high-quality meat. Meat quality, an important economic trait in the poultry industry, is primarily determined by multiple factors including intramuscular fat (IMF) content, pH value, meat color, drip loss, cooking loss and tenderness, which are closely associated with the fatty acid (FA) composition of skeletal muscle (Barbut, 2014; Hou et al., 2023; Chen et al., 2024). Variations in muscle FA composition not only have important effects on firmness of the fat in meat, especially subcutaneous and intermuscular fat, but also on the IMF content (Wood et al., 2004). IMF content is positively correlated with saturated fatty acids (SFA), while fat deposition and IMF content have a negative genetic correlation with polyunsaturated fatty acids (PUFAs) levels (Zhang et al., 2019; Yin et al., 2023). Therefore, muscle FA composition is a critical determinant of fat quality and is closely linked to the quality of chicken.

Muscle FA composition is regulated by a combination of heredity, diet, and gut microbiota (Yin et al., 2023). Recently, the gut microbiota has been considered an important modulator of complicated traits in livestock. Mounting evidence has indicated that the gut microbiota regulate fat deposition in humans (Turnbaugh et al., 2009; Serena et al., 2018), mice (Bäckhed et al., 2004; Samuel and Gordon, 2006; Turnbaugh et al., 2006), and domestic animals (Ding et al., 2016; Wen et al., 2019; Yin et al., 2023; Yang et al., 2025) by metabolite-host interactions. The gut microbiota undergoes a process of mutual selection and adaptive co-evolution with the host, which may be associated with their diverse phenotypes (Ma et al., 2022; Song et al., 2022). Notably, some of the gut microbiota-derived metabolites have been identified to regulate lipid metabolism and fat deposition, including short-chain fatty acids (Nogal et al., 2021), trimethylamine-N-oxide (Yoo et al., 2021), secondary bile acids (Zha et al., 2024), and branched-chain amino acids (Yang et al., 2025).

Here, we observed significant differences in meat quality traits between Daheng chickens (DHC, commercial fast-growing hybrids) and Luning chickens (LNC, slow-growing native chicken). However, it remains unclear whether and which gut microbiota contribute to the meat quality differences between DHC and LNC. Therefore, in the present study, we systematically evaluated the muscle FA profiles, variations in the gut microbiota, microbial metabolites, serum metabolites and muscle metabolites, respectively. The objective of the study was to identify the contribution of the gut microbiota to the muscle FA composition and provide evidence on how the gut microbiota promote fat deposition in the skeletal muscle of chicken.

## 2 Materials and methods

### 2.1 Animals and samples collection

A total of 80 one-day-old male DHCs (8 replicates with 5 chickens per replicate) and LNCs (8 replicates with 5 chicks per replicate) were randomly obtained and raised over a 150-day experimental period. DHCs were provided by Daheng Poultry Breeding Co., Ltd. (Chengdu, Sichuan Province, China), and LNCs were obtained from the Luning Chicken Genetic Resources Breeding Farm (Liangshan Yi Autonomous Prefecture, Sichuan Province, China). The microclimate conditions were strictly regulated according to standard indigenous chicken management guidelines. The brooding temperature was initially set to 33–35 °C during days 1–3, then gradually decreased by 2–3 °C per week, and maintained at a constant ambient temperature of 22 ± 1 °C from day 28 through day 150. Relative humidity (RH) was maintained at 65 -70% during the first week, and subsequently kept between 50 and 60% for the remainder of the experimental period. The lighting schedule was set to 23 h light: 1 h dark (23 L:1D) for days 1–3, 18 L:6D for days 4–42, and 16 L:8D from day 43 until slaughter at 150 days of age. Birds were fed standard basal diets formulated in three feeding phases, and the detailed ingredients and composition are presented in Supplementary Table S1. All chickens were given unrestricted access to their respective diets and water, and vaccination procedures were performed according to standard commercial protocols throughout the experimental period. Chickens were housed in three-tier stainless steel battery cages with individual cage dimensions of 100 cm in length, 60 cm in width, and 40 cm in height. During days 1–7, each cage accommodated 20 chicks, from days 8–14, the stocking density was adjusted to 10 birds per cage. During days 15–42, each cage accommodated 5 chickens and the density was adjusted to 2 birds per cage to ensure animal welfare until slaughter. To ensure independent biological replication, exactly one representative chicken was randomly selected from each of the 8 replicate pens per breed (totaling 8 individual chickens per breed) for the subsequent meat quality traits measurements, metagenomic sequencing, and multi-compartment metabolomics assessments. Prior to slaughter, approximately 2.0 mL of blood was collected from the brachial vein and stored in centrifuge tubes for serum isolation. All chickens were humanely sacrificed by exsanguination, and the left breast muscle, leg muscle, cecum digesta, and serum were immediately collected for preservation and further analysis.

### 2.2 Meat quality traits measurement

Eight chickens from each breed were selected for the meat quality traits measurement. The post-mortem pH of muscle at 45 min and 24 h was measured using a portable pH meter (Tetso 205, Testo SE & Co KGaA, Titisee-Neustadt, Germany) at three different locations. Meat color parameters including lightness (L∗), redness (a∗) and yellowness (b∗) were determined in triplicate by a CR-410 hand-held colorimeter (Konica Minolta, Tokyo, Japan). Drip loss and cooking loss measurement were performed at 24 h post-mortem according to Luo’s study (Chen et al., 2024). The IMF content was determined with petroleum ether using a Soxtec Extraction method. The histological characteristics of muscle were evaluated by hematoxylin and eosin (H&E) staining as described in our previous study (Pan et al., 2022). Shear force was measured by a texture analyzer (TA. XTC-18, Baosheng, Shanghai, China) at a speed of 200 mm/min. The mean value of three replicate was used as the final result for all the meat quality traits.

### 2.3 Metagenomic sequencing for gut microbiota

0.2 g of cecal contents of DHC and LNC were used to extract total genomic DNA with the FastPure Stool DNA Isolation Kit (MJYH, shanghai, China) according to manufacturer’s instructions. Concentration and purity of extracted DNA was determined with SynergyHTX and NanoDrop2000, respectively. DNA quality was checked on 1% agarose gel.

For library preparation, the extracted genomic was sheared into fragments with an average size of 350 bp utilizing a Covaris M220 focused ultrasonicator (Gene Company Limited, Shanghai, China). Paired-end sequencing libraries were subsequently generated using the NEXTFLEX Rapid DNA-Seq Kit (Bioo Scientific, Austin, TX, USA). High-throughput metagenomic sequencing was then executed on the Illumina NovaSeq X Plus platform (Illumina Inc., San Diego, CA, USA).

The raw sequencing reads were trimmed of adapters, and low-quality reads (length<50 bp or with average quality value <20) were removed by fastp (v0.20.0) (Chen et al., 2018). To eliminate host genomic contamination, the quality-filtered reads were mapped against the chicken reference genome (*Gallus gallus* GRCg6a) using BWA (v0.7.17) (Li and Durbin, 2009), and all high-scoring host-aligned reads were discarded. *De novo* assembly of the clean, non-host reads was performed using MEGAHIT (v1.1.2) (Li et al., 2015). Contigs with a minimum length of 300 bp were selected as the final assembling result. Open reading frames (ORFs) were predicted on the assembled contigs leveraging Prodigal (v2.6.3) (Hyatt et al., 2010), and a length ≥ 100 bp ORFs were retrieved. A non-redundant gene catalog was subsequently established using CD-HIT (v4.7.0) (Fu et al., 2012) based on a threshold of 90% sequence identity and coverage. To determine relative gene abundance across individual samples, clean reads were back-mapped to the gene catalog via SOAPaligner (v2.2.1) (Li et al., 2008) with a 95% identity criterion.

Taxonomic assignment was conducted by aligning the non-redundant genes against the NCBI non-redundant (NR) protein database using DIAMOND (v2.0.13) (Buchfink et al., 2015) with an E-value cutoff of 1e^−5^. Similarly, functional profiling, including classification into the Gene Ontology (GO) and Kyoto Encyclopedia of Genes and Genomes (KEGG) of non-redundant genes was obtained. Based on the taxonomic and functional annotation and the abundance profile of non-redundant genes, the differential analysis was carried out at each taxonomic, functional, or gene-wise level by Kruskal-Wallis test.

### 2.4 GC–MS targeted metabolomics for muscle fatty acids

To establish the standard curves, a stock solution (A) containing 51 fatty acid methyl esters (FAMEs) was prepared by dissolving 100 mg of the FAME mixture in 1 mL of dichloromethane. A 100 μL aliquot of solution A was then diluted with 900 μL of dichloromethane to yield an intermediate solution (B), which was subsequently serially diluted to generate six working standard levels (L1-L6).

For sample preparation, 50 mg of muscle tissue was homogenized with 1 mL of a chloroform/methanol mixture (1:1, v/v) using a Wonbio-96c frozen tissue grinder (Wanbo, Shanghai, China) at 50 Hz for 3 min, followed by ultrasonic extraction at a low temperature for 15 min. After incubating at −20 °C for 15 min, the homogenate was subjected to centrifugation at 13,000 rpm for 10 min (4 °C). A 500 μL aliquot of the resulting supernatant was evaporated to dryness under a stream of nitrogen. For methylation, the residue was reconstituted in 0.2 mL of 0.5 mol/L sodium hydroxide-methanol solution, sonicated at 4 °C for 10 min, and then incubated in a water bath at 60 °C for 30 min. Upon cooling, 0.5 mL of n-hexane was introduced, and the mixture was centrifuged again (13,000 rpm, 10 min, 4 °C). Finally, a 100 μL portion of the upper n-hexane layer was collected for subsequent GC–MS analysis.

Chromatographic separation was conducted on an Agilent 8890B gas chromatograph configured with an Agilent 5977B mass selective detector (Agilent, Santa Clara, CA, USA) at Majorbio Bio-Pharm Technology Co. Ltd. (Shanghai, China). Target analytes were separated using an Agilent CP-Sil88 for FAME capillary column (100 m × 0.25 mm × 0.20 μm) with high-purity helium (99.999%) as the carrier gas at a constant flow rate of 1 mL/min. The oven temperature program was initiated at 80 °C, increased to 180 °C at 8 °C/min, ramped to 220 °C at 4 °C/min, further elevated to 230 °C at 2 °C/min (held for 13.5 min) and finally maintained at 235 °C for 2 min. A 1 μL sample was injected in split mode (50:1) with an inlet temperature maintained at 260 °C. Mass spectrometry was operated in selected ion monitoring (SIM) mode, with the ion source and quadrupole temperatures set to 230 °C and 150 °C, respectively.

Raw GC–MS data were processed using MassHunter quantitative software (v10.0.707.0). Automated identification and peak integration of all ion fragments were executed based on default parameters, followed by meticulous manual verification. Fatty acid concentrations in muscle samples were quantified utilizing the linear regression equations derived from the standard curves.

### 2.5 LC–MS untargeted metabolomics for cecal content, serum, and muscle

For metabolite extraction, 50 mg of each sample (cecal content, serum, or muscle) was treated with 400 μL of a pre-cooled methanol/water mixture (4:1, v/v). Muscle and cecal content samples were thoroughly homogenized using a tissue grinder at 50 Hz for 6 min, while serum samples were vigorously vortexed. The mixtures were then subjected to low-temperature ultrasonic extraction at 40 Hz for 30 min. To facilitate protein precipitation, the extracts were incubated at −20 °C for 30 min, followed by centrifugation at 13,000 rpm for 15 min (4 °C). The resulting clarified supernatants were subsequently transferred to autosampler vials for LC–MS/MS analysis.

Chromatographic separation was achieved on a Vanquish UHPLC system (Thermo Fisher Scientific, Waltham, MA, USA) equipped with an ACQUITY UPLC HSS T3 column (100 mm × 2.1 mm, 1.8 μm; Waters, Milford, MA, USA) maintained at 40 °C, running at a constant flow rate of 0.40 mL/min. The mobile phase architecture comprised (A) 0.1% formic acid in water/acetonitrile (95:5, v/v) and (B) 0.1% formic acid in acetonitrile/isopropanol/water (47.5:47.5:5, v/v/v). Mass spectrometry was performed on a Q Exactive HF-X Hybrid Quadrupole-Orbitrap mass spectrometer (Thermo Fisher Scientific) operating in both positive and negative electrospray ionization (ESI) modes. The source parameters were optimized as follows: source temperature, 400 °C; sheath gas flow rate, 40 arb; auxiliary gas flow rate, 10 arb; and ion-spray voltage floating (ISVF), 3,500 V for positive mode and −2,800 V for negative mode. Data acquisition was executed in data-dependent acquisition (DDA) mode across a scanning range of 70–1,050 m/z. Full MS and MS/MS resolutions were configured at 70,000 and 17,500, respectively, with fragmentation induced by a rolling normalized collision energy (NCE) of 20–40-60 V.

The acquired LC–MS raw profiles were processed utilizing Progenesis QI software (v3.0, Waters Corporation, Milford, MA, USA) for peak alignment, picking, and normalization, yielding a three-dimensional data matrix comprising retention times, m/z pairs, and normalized intensities. Metabolite identification was performed by matching the tandem mass spectra against public repositories, specifically the Human Metabolome Database (HMDB) and the METLIN database.

### 2.6 Mediation analysis

To elucidate the potential causal mechanisms through which the gut microbiota influences muscle fatty acid profiles via altered metabolites, a counterfactual framework-based mediation analysis was executed utilizing the “mediation” package in R (The R scripts and mediation models were uploaded in the Supplementary files) (Rijnhart et al., 2021; Zhou et al., 2025). Within this framework, linear regression models were constructed to estimate the average causal mediation effects (ACME), average direct effects (ADE), and total effects for each target variable. The proportion of the effect mediated (Prop) was subsequently quantified as the ratio of the estimated indirect effect to the total effect. *p*-values derived from the mediation analysis were evaluated both with and without multiple testing correction (Benjamini–Hochberg, BH). If no mediation pathways achieved statistical significance following multiple testing correction, those identified using uncorrected *p*-values were retained for further exploratory analysis. Statistically robust pathways fulfilling the criteria of both *P*_ACME_ < 0.05, and *P*_Prop_ < 0.05 were selectively visualized using a Sankey diagram.

### 2.7 Statistical analysis

The data was presented as mean ± SEM. Statistical significances between DHC and LNC were determined by Student’s t-test by IBM SPSS Statistics 26.0 software (v26.0), and *p* value < 0.05 was considered significant. To explore the interconnectedness among meat quality traits, muscle fatty acids, gut microbiota, and metabolic profiles, Spearman correlation analysis and Mantel test (The R scripts were uploaded in the Supplementary files) were performed leveraging the “ggcor” package in R. *p* < 0.05 was considered statistically significant.

## 3 Results

### 3.1 Meat quality traits of breast and leg muscle in Daheng and Luning chicken

**Figure 1:**
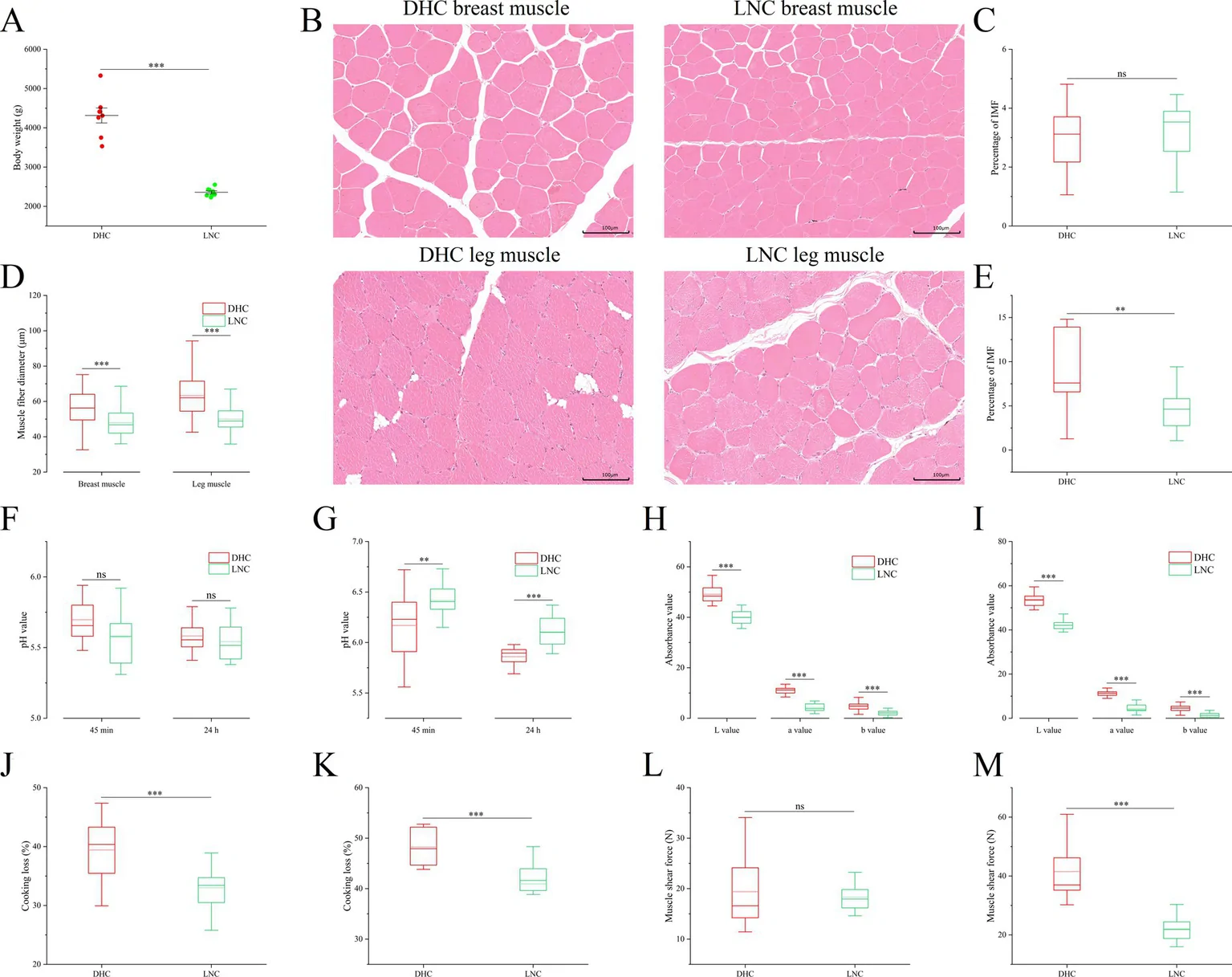
The meat quality traits of breast and leg muscle between Daheng (DHC) and Luning (LNC) chicken. **(A)** The body weight of DHC and LNC chicken. **(B)** H&E staining of breast and leg muscle cross-section in between DHC and LNC. Bars = 100 μm. **(C)** Percentage of IMF in breast muscle. **(D)** Muscle fiber diameter of breast and leg muscle. **(E)** Percentage of IMF in leg muscle. **(F)** pH value of breast muscle. **(G)** pH value of leg muscle. **(H)** Meat color of breast muscle. **(I)** Meat color of leg muscle. **(J)** Cooking loss of breast muscle. **(K)** Cooking loss of leg muscle. **(L)** Shear force of breast muscle. **(M)** Shear force of leg muscle.

The meat quality traits of breast and leg muscle between DHC and LNC were presented in Figure 1. The live body weight of DHC was higher than that of LNC at 150 days (Figure 1A). H&E staining results showed the muscle fiber diameter of both breast and leg muscle of DHC were larger than that in LNC (Figures 1B,D). Additionally, The IMF content in the DHC leg muscle was significantly higher than that in the LNC muscle (Figure 1C), while there was no significant difference in the breast muscle (Figure 1E). No significant differences were observed in the pH values of breast muscle at 45 min and 24 h post slaughter between DHC and LNC (Figure 1F), but there was significant difference observed in the pH values of leg muscle (Figure 1G). Compared to LNC, the breast muscle of DHC at 45 min and 24 h exhibited higher brightness (L*), redness (a*) and yellowness (b*) (Figures 1H,I). Furthermore, the breast muscle of DHC had higher cooking loss, while no significant difference in drip loss and shear force. (Figures 1J,K) For the leg muscle, both cooking loss and shear force were significantly higher in DHC than in LNC (Figures 1L,M).

**Figure 2:**
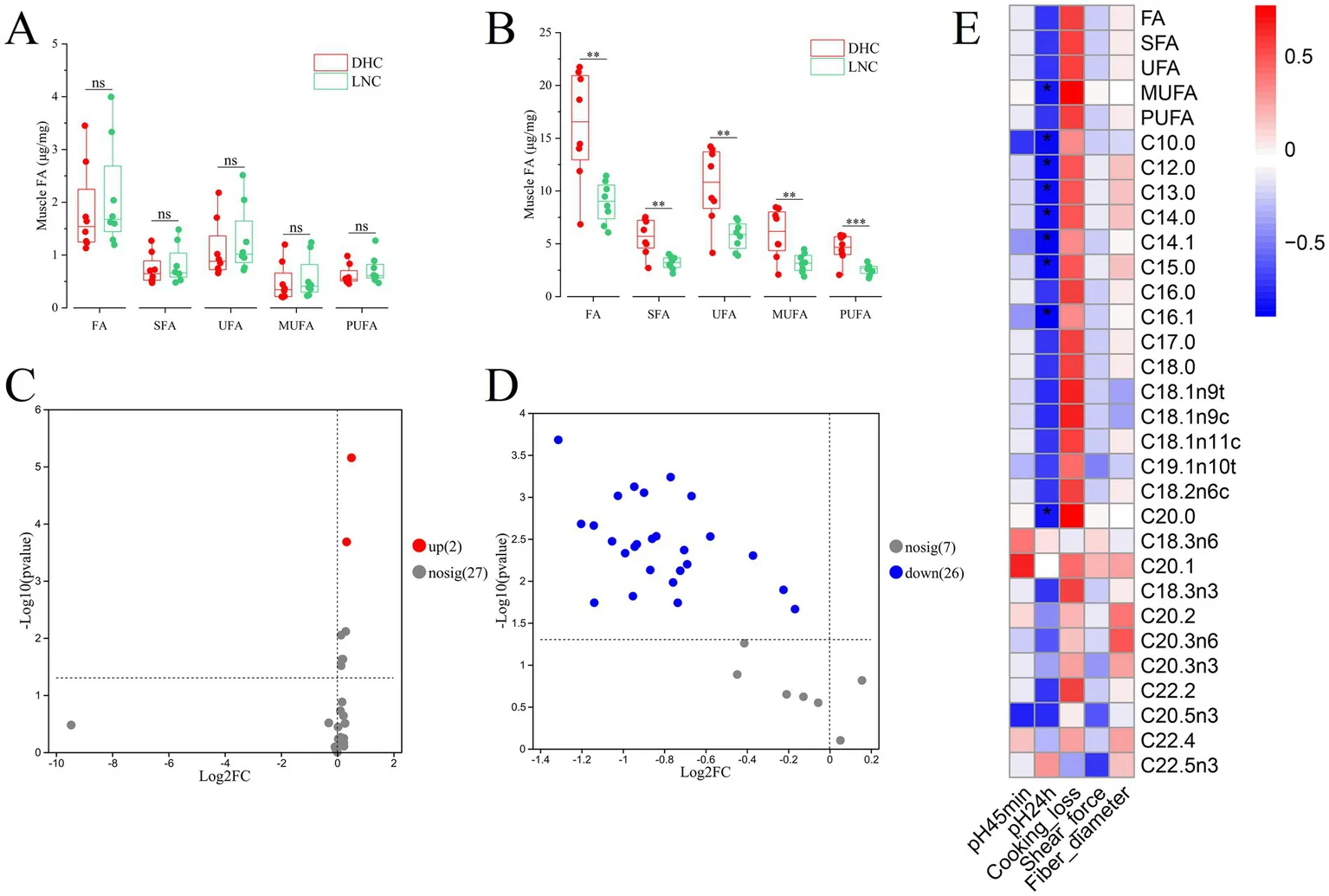
Muscle FAs were differentially expressed between DHC and LNC. **(A)** Muscle FA concentrations of breast muscle. **(B)** Muscle FA concentrations of leg muscle. **(C)** Volcano plot of 2 differentially expressed FAs in breast muscle. **(D)** Volcano plot of 26 differentially expressed FAs in leg muscle. **(E)** Pearson correlation analysis between FAs and meat quality traits in the leg muscle of DHC. ^*^p < 0.05, ^**^p < 0.01.

Based on the results obtained from the targeted metabolomic sequencing of muscle medium and long-chain fatty acids (FA), The total FAs, saturated fatty acids (SFAs), unsaturated fatty acids (UFAs), monounsaturated fatty acids (MUFAs), and polyunsaturated fatty acids (PUFAs) concentrations did not change in the breast muscle of two chicken breeds (Figure 2A). In contrast, The FAs, SFAs, UFAs, MUFAs, and PUFAs were all significantly enriched in the leg muscle of DHC compared with LNC (Figure 2B). In detail, the concentrations of 2 PUFAs were significantly increased in the LNC breast muscle, namely C22:5n6 and C22:6n3 (Figure 2C; Supplementary Table S2). The concentrations of 26 FAs were significantly increased in the DHC leg muscle, including 9 SFAs (C10:0, C12:0, C13:0, C14:0, C15:0, C16:0, C17:0, C18:0, C20:0), 7 MUFAs (C14:1, C16:1, C18:1n9t, C18:1n9c, C18:1n11c, C19:1n10t, C20:1), and 10 PUFAs (C18:2n6c, C18:3n6, C18:3n3, C20:2, C20:3n6, C20:3n3, C22:2, C20:5n3, C22:4, C22:5n3) (Figure 2D; Supplementary Table S2). Notably, DHC accumulated significantly elevated levels of key flavor-associated MUFAs (C18:1n9c) as well as functional omega-3 (n-3) PUFAs including C20:5n3 and C22:5n3. Correlation analysis was performed to explore the potential relationship between the differential FAs and meat quality traits in the leg muscle of DHC, which identified seven negative associations for C10:0 (pH45min and pH24h), C12:0 (pH24h), C13:0 (pH24h), C14:1 (pH24h), C16:1 (pH24h) and C20:5n3 (pH45min) (Figure 2E).

### 3.2 Variations in the cecal gut microbiota related to meat quality traits

**Figure 3:**
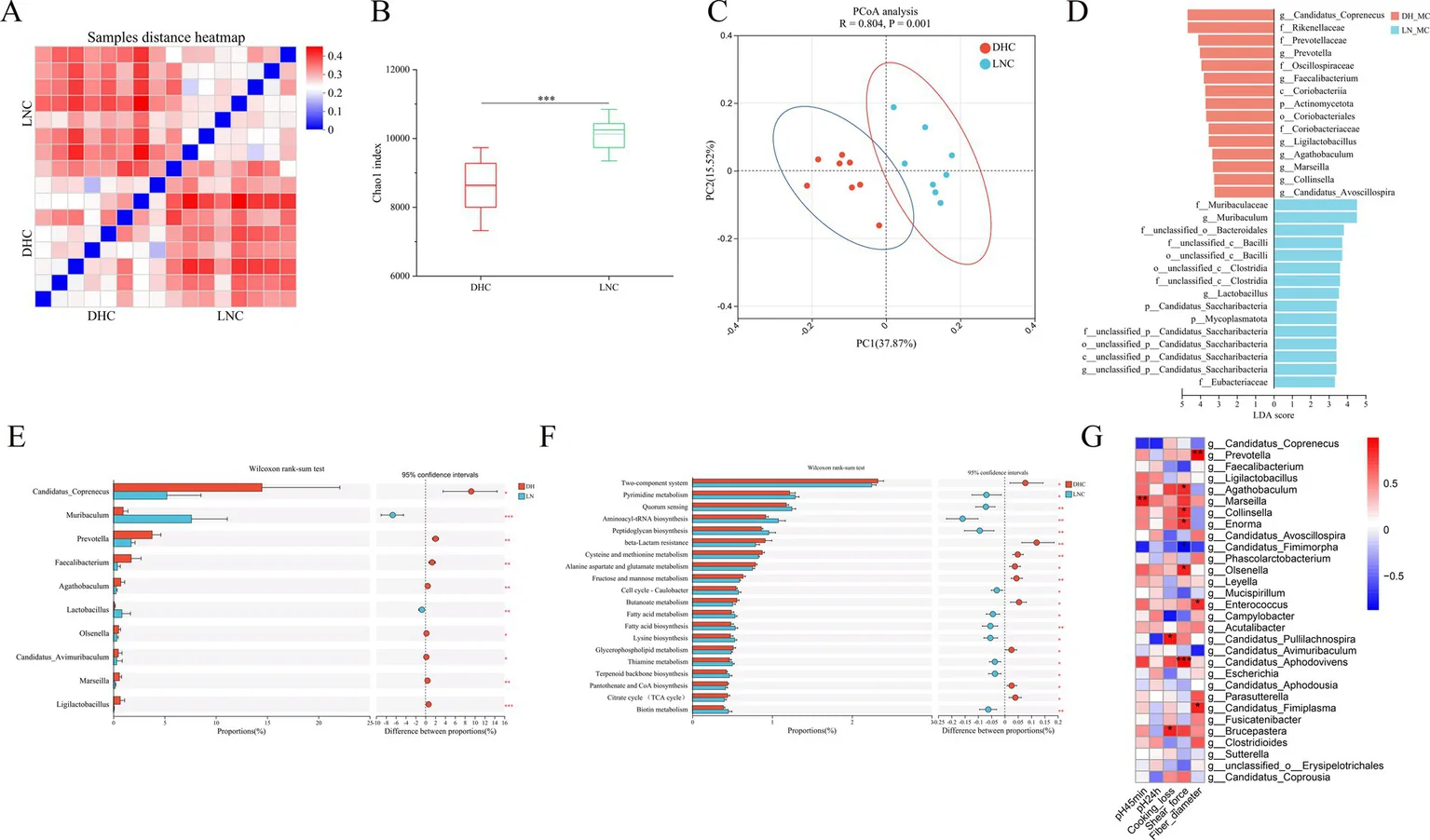
The difference of gut microbial function and metabolic function between DHC and LNC and correlated meat quality traits. **(A)** The distance between samples. **(B)** Chao1 indices of the cecal microbiome. **(C)** PCoA of the Bray-Curtis distances of the cecal microbiome. **(D)** Line discriminant analysis (LDA) effect size (LEfSe) analysis. The default parameters used were LDA score >2 and *p* < 0.05. **(E)** Significant changes in abundance of cecal microbes at the genus levels. **(F)** Functional enrichment analysis of KEGG pathways related to changes in cecal microbiota. **(G)** Spearman correlation analysis between the dominant bacteria and meat quality traits in the leg muscle of DHC. ^*^p < 0.05, ^**^p < 0.01, ^***^p < 0.001.

We performed metagenomic sequencing to analyze the cecal gut microbial profiles of two chicken breeds, with an average of 36,435,640 optimized reads per sample generated for splicing and assembly. Distance heatmap analysis showed that distance differences were observed in DHC and LNC (Figure 3A). The *α*-diversity analysis showed significant decrease in the Chao1 indices in DHC compared with LNC (Figure 3B). The *β*-diversity analysis also showed significant differences in the cecal microbial community structure between DHC and LNC (Figure 3C). Moreover, the dominant bacteria at the genus level were predicted by LEfSe analysis (using an LDA score threshold of 2.0) and Wilcoxon rank-sum test. The result demonstrated that *Candidatus Coprenecus*, *Prevotella*, *Faecalibacterium*, *Ligilactobacillus*, *Agathobaculum* and *Marseilla* were found to be predominant in the DHC (Figures 3D,E; Supplementary Table S3). The microbial function of the KEGG pathways revealed that cysteine and methionine metabolism, pantothenate and CoA biosynthesis were enriched in DHC (Figure 3F). We further performed Spearman correlation analyses between the dominant bacteria at the genus level and meat quality traits in the leg muscle of DHC. Of the 30 dominant bacteria (LDA > 2), twelve were markedly correlated with the meat quality traits (Figure 3G), including the differentially expressed *Prevotella*, *Agathobaculum*, *Marseilla*, *Collinsella*, *Enorma*, *Candidatus*_Avoscillospira, *Olsenella*, *Enterococcus*, *Candidatus_Pullilachnospira*, *Candidatus_Aphodovivens*, *Candidatus*_Fimiplasma, and *Brucepastera*. These results revealed distinct gut bacterial profiles between DHC and LNC, which might be closely associated with the meat quality traits of chickens.

### 3.3 Metabolome profiling of the cecum, serum, and muscle differed between DHC and LNC

**Figure 4:**
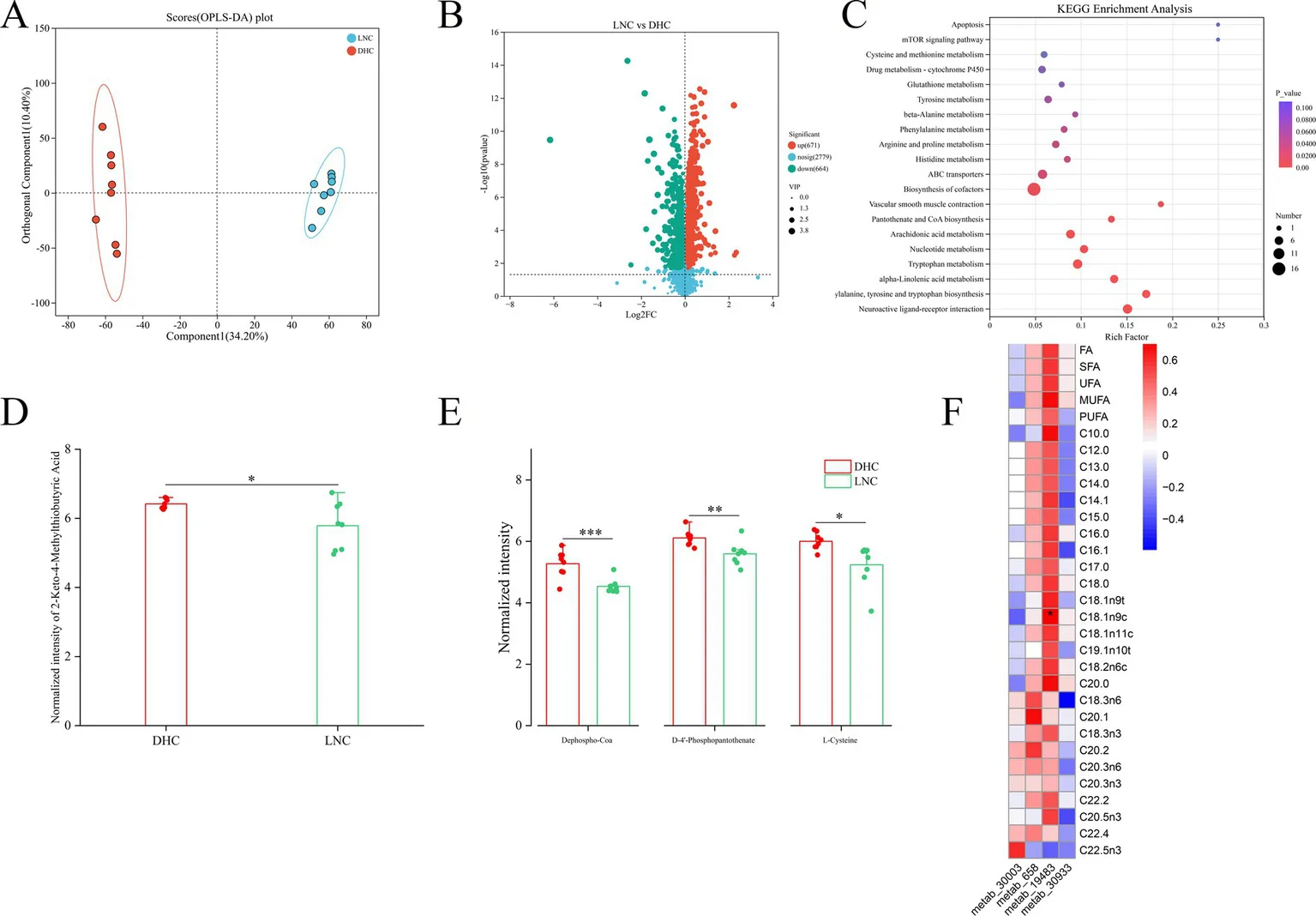
The significantly altered metabolites in cecum were related to FAs. **(A)** OPLS-DA plots of DHC and LNC groups. **(B)** Volcano plots of the differential metabolites in cecum. **(C)** KEGG pathway enrichment of the differential metabolites. **(D)** The different metabolite enriched in cysteine and methionine metabolism pathway. **(E)** The different metabolite enriched in pantothenate and CoA biosynthesis pathway. **(F)** Spearman correlation analysis between the significantly altered metabolites and FAs in DHC. ^*^p < 0.05.

A nontargeted LC–MS-based metabolomics approach was employed to investigate the differential metabolites between DHC and LNC, and explore their connections with the cecal microbiome. The OPLS-DA model revealed a distinct clustering of DHC and LNC, indicating clear metabolic differences between the cecum of two chicken breeds (Figure 4A; Supplementary Figure S1). Among the 4,114 detected metabolites, 671 were significantly upregulated, and 664 were significantly downregulated in LNC (*p* < 0.05) (Figure 4B; Supplementary Table S4). KEGG pathway analysis revealed that cysteine and methionine metabolism and pantothenate and CoA biosynthesis pathways were significantly enriched, involving multiple metabolites including 2-keto-4-methylthiobutyric acid, Dephospho-coa, D-4’-Phosphopantothenate, and L-cysteine (Figures 4C–E). Spearman correlations analysis between four circulating differential metabolites and 26 significantly increased FAs in the DHC leg muscle showed that D-4’-Phosphopantothenate was significantly positively correlated with C10:0, C14:1 and C16:1 (*p* < 0.05) (Figure 4F).

**Figure 5:**
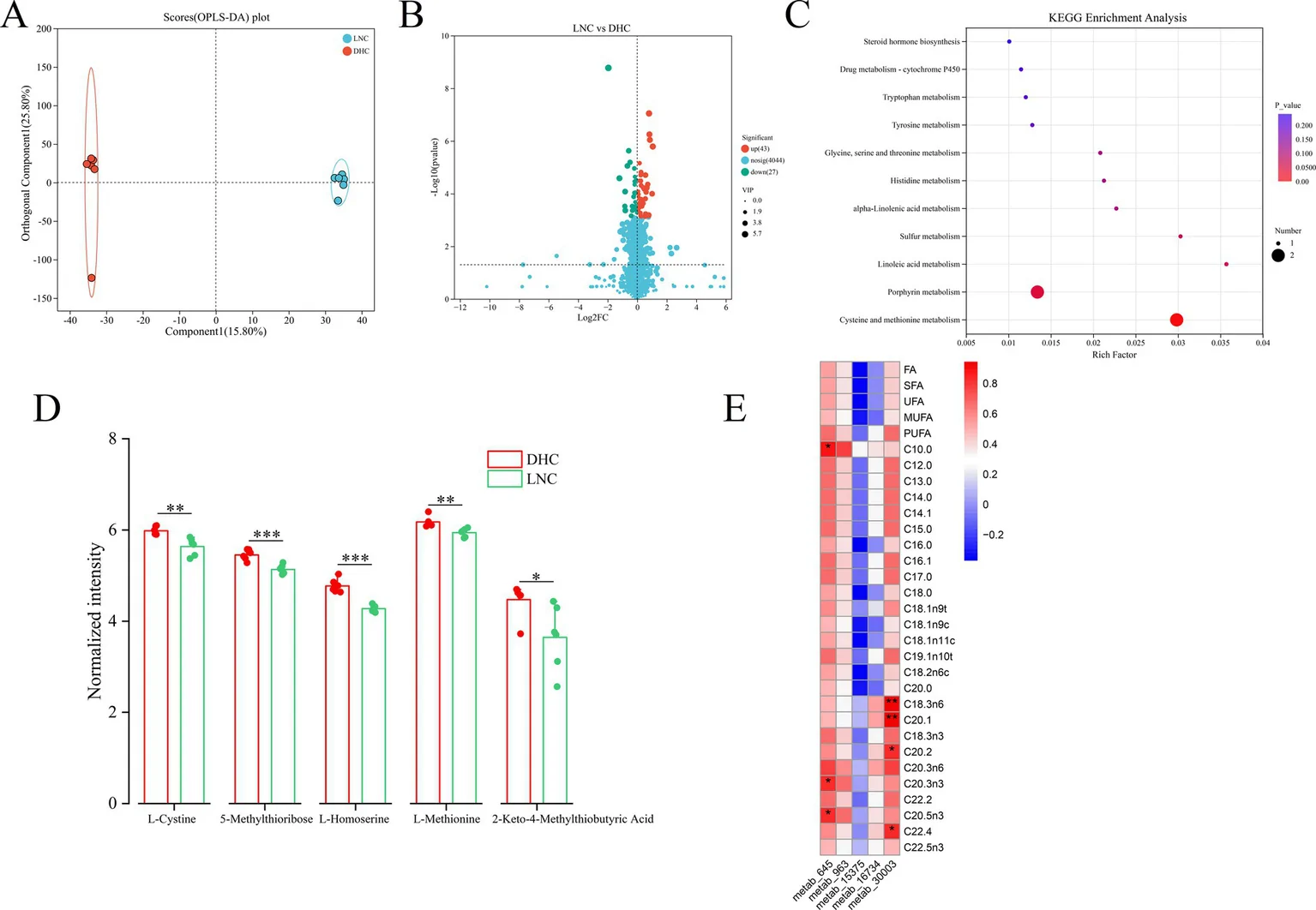
The significantly altered metabolites in serum were related to FAs. **(A)** OPLS-DA plots of DHC and LNC groups. **(B)** Volcano plots of the differential metabolites in serum. **(C)** KEGG pathway enrichment of the differential metabolites. **(D)** The different metabolite enriched in cysteine and methionine metabolism pathway. **(E)** Spearman correlation analysis between the significantly altered metabolites and FAs in DHC. ^*^p < 0.05, ^**^p < 0.01.

The OPLS-DA model showed a distinct separation between the serum metabolomes of DHC and LNC (Figure 5A; Supplementary Figure S2). Among 4,114 measured metabolites, 43 were significantly different (*p* < 0.05) (Figure 5B; Supplementary Table S5). KEGG pathway analysis indicated significant alterations in multiple metabolites involved in cysteine and methionine metabolism, specifically L-Cystine, 5-methylthioribose, L-homoserine, L-methionine, and 2-keto-4-methylthiobutyric acid (Figures 5C,D). Spearman’s correlation analysis revealed correlations between three circulating differential metabolites and 26 significantly increased FAs in the DHC leg muscle (Figure 5E). Specifically, L-cystine was significantly positively correlated with C10:0, C20:3n3 and C20:5n3 (*p* < 0.05), and 2-keto-4-methylthiobutyric acid was significantly positively correlated with C18:3n6, C20:1, C20:2, and C22:2 (*p* < 0.05).

**Figure 6:**
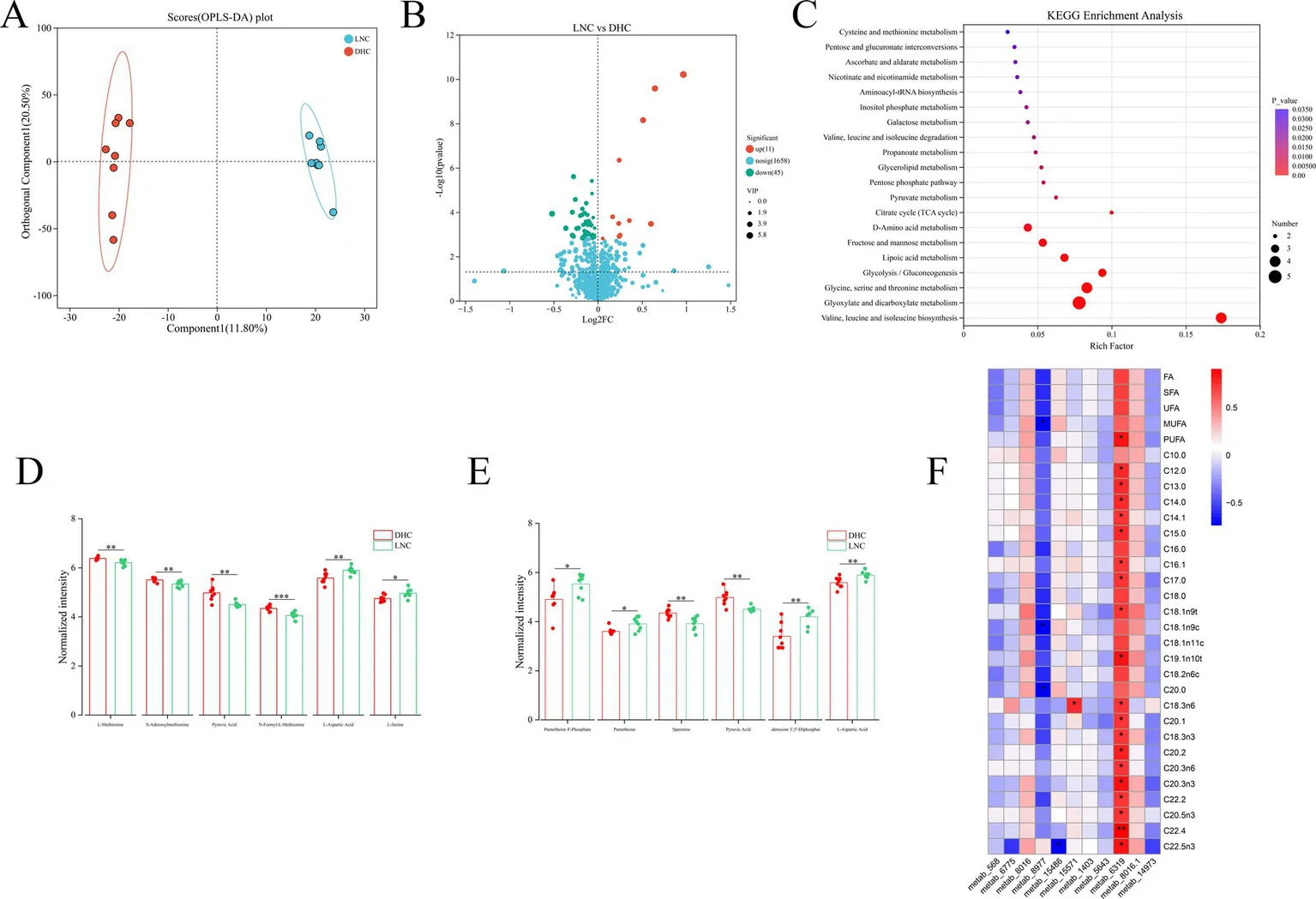
The significantly altered metabolites in leg muscle were related to FAs **(A)** OPLS-DA plots of DHC and LNC groups. **(B)** Volcano plots of the differential metabolites in leg muscle. **(C)** KEGG pathway enrichment of the differential metabolites. **(D)** The different metabolite enriched in cysteine and methionine metabolism pathway. **(E)** The different metabolite enriched in pantothenate and CoA biosynthesis pathway. **(F)** Spearman correlation analysis between the significantly altered metabolites and FAs in DHC. ^*^p < 0.05, ^**^p < 0.01.

The OPLS-DA score plot also showed a distinct clustering in the leg muscle metabolome between DHC and LNC (Figure 6A; Supplementary Figure S3). Among 1,714 measured metabolites, 11 were significantly upregulated, and 45 were significantly downregulated in LNC (*p* < 0.05) (Figure 6B; Supplementary Table S6). KEGG pathway analysis further confirmed significant enrichment in cysteine and methionine metabolism as well as pantothenate and CoA biosynthesis (Figure 6C). Within cysteine and methionine metabolism pathway, L-methionine, S-adenosylmethionine, Pyruvic acid and N-formyl-L-methionine were significantly downregulated in DHC, while L-aspartic acid and L-serine were significantly upregulated. In the pantothenate and CoA biosynthesis pathway, Spermine and Pyruvic acid were significantly downregulated while Pantetheine 4′-phosphate, Pantetheine, Adenosine 3′,5′-diphosphate, and L-aspartic acid were significantly upregulated (Figures 6D,E). Analysis of Spearman correlation between 10 circulating differential metabolites and 26 significantly increased FAs in the DHC leg muscle revealed that N-formyl-L-methionine negatively correlated with C18:1n9c, C20:0 and total MUFAs (*p* < 0.05), L-aspartic acid negatively correlated with C22:5n3 (*p* < 0.05), and L-serine positively correlated with C18:3n6 (*p* < 0.05), respectively (Figure 6F). Interestingly, Spermine was significantly positively correlated with C12:0, C13:0, C14:0, C14:1, C15:0, C16:1, C17:0, C18:1n9t, C19:1n10t, C18:3n6, C20:1, C18:3n3, C20:2, C20:3n6, C20:3n3, C22:2, C20:5n3, C22:4, C22:5n3, and PUFA in the DHC leg muscle (Figure 6F).

### 3.4 The gut microbiome is associated with muscle fatty acid levels via intermediate metabolites

**Figure 7:**
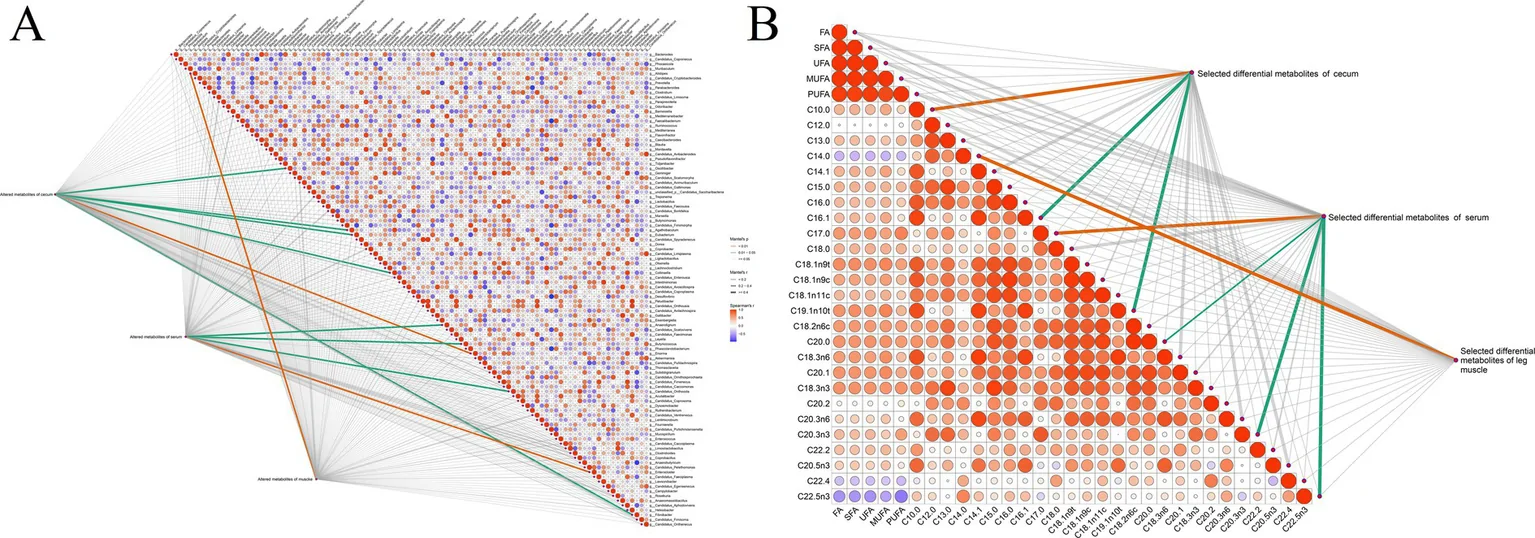
Correlation analysis among the gut microbiome, metabolites and FAs. **(A)** The correlations among the gut microbiome, cecum, serum and muscle altered metabolites. **(B)** The correlations among the muscle fatty acids, cecum, serum and muscle differential metabolites of cysteine and methionine metabolism and pantothenate and CoA biosynthesis pathway.

To unravel the mechanistic pathways through which the dominant gut microbiota in DHC modulates muscle FA profiles, and to define the role of specific metabolites in the gut-muscle axis, we initially evaluated the connectivity between key microbial taxa and altered metabolomes in the cecum, serum, and muscle using Spearman correlation and Mantel tests. The analysis revealed that the *Oscillibacter*, *Agathobaculum*, *Eubacterium*, *Collinsella*, *Enorma*, *Candidatus_Onthenecus*, *Anaerotignum*, *Butyricicoccus*, *Candidatus_Onthocola*, *Enterocloster*, *Fibrobacter*, and *Alistipes* were significantly correlated with the differential metabolites across cecum, serum and muscle (Figure 7A). Given these strong associations, we hypothesized that these metabolic intermediates may act as molecular conduits in the gut-muscle crosstalk, potentially mediating the microbial influence on downstream FA deposition. To test this hypothesis, we specifically focused on the differential metabolites enriched in “cysteine and methionine metabolism” as well as “pantothenate and CoA biosynthesis” pathways. Correlation matrices and Mantel tests demonstrated a dense network of significant associations between muscle FAs and these pathway-specific metabolites (Figure 7B). This co-variation underscores the potential regulatory role that these two metabolic pathways play in governing muscle FA deposition. Collectively, these insights indicate that the identified metabolites function as pivotal bridges within the gut-muscle axis, profiling the muscle FA landscape.

**Figure 8:**
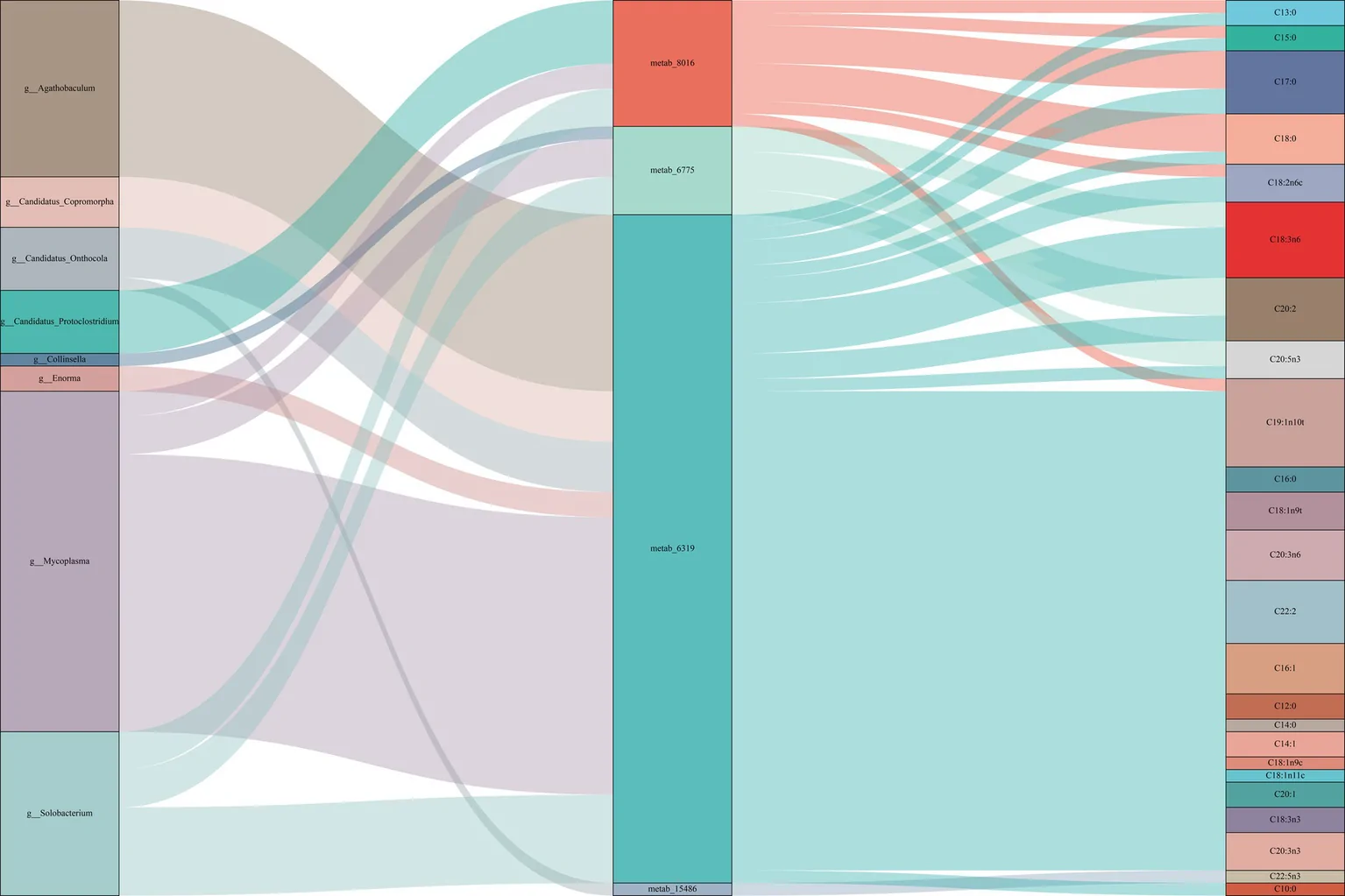
The mediation role of metabolites in the gut microbiota-muscle axis.

Subsequently, mediation analysis was deployed to integrationally model potential directional paths among the aforementioned gut microbiota, metabolic intermediates, and muscle FAs (Figure 8; Supplementary Table S7). Based on the results shown in Figures 3A, 7A, 8, the *Agathobaculum*-*Collinsella*-*Enorma* consortium was selected as the core genera. Intriguingly, the identified mediation pathways were predominantly linked to microbial co-variation with FAs, centered around key candidate molecular nodes: Pyruvic Acid (metab_8016), S-Adenosylmethionine (metab_6775), and Spermine (metab_6319). These statistical networks support our hypothesis, indicating that these specific metabolites may operate as intermediate nodes within the gut-muscle axis that ultimately affected the FA composition in chicken muscle.

## 4 Discussion

Driven by escalating consumer demand for premium poultry products, optimizing meat quality and nutritional profiles has emerged as a primary objective for both livestock researchers and the poultry industry. In this study, we conducted a comparative evaluation between a commercial, fast-growing hybrid breed (DHC) and a traditional, slow-growing native breed (LNC). Our phenotypic profiling unveiled distinct variations in meat quality traits between the two breeds. Intriguingly, the leg muscle of DHC exhibited an enriched accumulation of total FAs, SFAs, MUFAs, and PUFAs. These lipophilic constituents were universally recognized as vital drivers of meat palatability and flavor attributes (Yin et al., 2023; Chen et al., 2024). Concurrently, high-throughput sequencing mapped out divergent structural landscapes within their respective gut microbiotas. Nevertheless, a pivotal question persists regarding how the localized dominant taxa in DHC orchestrate systemic crosstalk with distant skeletal muscles. Postulating that circulating metabolites function as molecular messengers bridging this gut-muscle axis, we profiled the metabolomes across the cecum, serum, and muscle compartments, which indeed confirmed distinctive, compartment-specific metabolic signatures between the two breeds. Ultimately, through integrative multi-omics modeling combined with statistical mediation analysis, we identified a systemic network: the gut microbiota is closed with intramuscular FA deposition, a process bridged by key intermediate metabolites heavily enriched in “cysteine and methionine metabolism” and “pantothenate and CoA biosynthesis” pathways.

The leg muscle of DHC demonstrated a selective accumulation of 26 distinct FAs, characterized by marked enrichments in specific MUFAs and PUFAs. Most prominently, DHC exhibited significantly elevated concentrations of C18:1n9c (Oleic acid), the predominant MUFA in avian skeletal muscle. Oleic acid serves as a critical determinant of meat organoleptic quality. During thermal processing, it undergoes lipid oxidation to generate key volatile aromatic aldehydes and ketones that impart the characteristic desirable flavor and savory aroma of cooked poultry (Fu et al., 2022). Furthermore, higher Oleic acid deposition is closely associated with superior muscle juiciness and tenderness (Wood et al., 2004). Simultaneously, DHC accumulated higher levels of essential n-3 PUFAs, including C20:5n3 (EPA) and C22:5n3 (DPA). n-3 PUFAs are well-recognized for their health-promoting properties in human nutrition, including anti-inflammatory activity and cardiovascular benefits (Mozaffarian and Wu, 2011). Previous comparative studies between slow-and fast-growing poultry lines reported trade-offs between lipid accretion and functional PUFA ratios (Sirri et al., 2011). In contrast, our current findings demonstrate that DHC simultaneously optimizes both flavor-precursor MUFAs and health-beneficial n-3 PUFAs. These findings indicate that DHC has an enhanced desaturation capacity, which likely supported by elevated substrate availability.

Undeniably, intramuscular FA deposition and composition are influenced by multi-faceted regulatory networks. Our findings indicate that these lipid profiles are, to a considerable extent, tightly linked with microbial characteristics. Specifically, we observed that the enrichment of distinctive dominant taxa in DHC—namely *Agathobaculum*, *Collinsella*, and *Enorma*, exhibited robust correlations with premium meat quality traits. Among these, *Agathobaculum* stands out as a premier butyrate-producing obligate anaerobe that plays a cornerstone role in reinforcing intestinal barrier integrity, quenching systemic inflammation, and fine-tuning host metabolic homeostasis (Siddiqui and Cresci, 2021; Pant et al., 2023; Nshanian et al., 2025). Emerging evidence underscores that butyrate serves as a potent systemic signaling ligand capable of triggering the AMPK and PPAR signaling cascades; this activation enhances mitochondrial biogenesis and efficiency, subsequently stimulating free fatty acid uptake and *β*-oxidation kinetics within distant myocytes (Song et al., 2025). In contrast to the direct butyrate-producing capacity of *Agathobaculum*, the metabolic end-products of *Collinsella* and *Enorma* fermentations are predominantly acetate and lactate. Circulating acetate has been documented to amplify cellular glucose import and accelerate skeletal muscle FA turnover via the phosphorylation of the AMPK axis (Maruta et al., 2016). Crucially, from an ecological standpoint, both acetate and lactate function as vital biosynthetic precursors that fuel secondary fermenters like *Agathobaculum* to synthesize butyrate via an intricate trophic cross-feeding mechanism (Louis et al., 2014). Taken together, these interconnected metabolic relays support the hypothesis that the *Agathobaculum*-*Collinsella*-*Enorma* consortium co-varies with muscle FA deposition and structural traits.

To decipher the functional underpinnings linking the dominant gut microbiota to phenotypic variations, we explored the molecular cascades aligned with intramuscular lipid deposition through a multi-omics lens. Our analyses revealed that metabolic fluctuations within the “cysteine and methionine metabolism” and “pantothenate and CoA biosynthesis” pathways were fundamentally tethered to muscle FA concentration. In poultry nutrition, targeted methionine supplementation is well-documented to modulate hepatic lipid homeostasis, accelerate muscle accretion, and refine meat quality traits (Yan et al., 2023; Wan et al., 2025). Aligned with these insights, our data indicated that the unique microbial configuration in DHC was associated with an elevated baseline of muscle FAs, tightly coupled with the enrichment of key sulfur-containing metabolites such as methionine. A parallel metabolic phenomenon was observed in Pekin ducks subjected to dietary methionine deprivation (Wu et al., 2022). Furthermore, nutritional restriction of cysteine has been shown to profoundly alter systemic adiposity, lipolytic networks, and cellular stress signaling—a cascade fundamentally precipitated by the depletion of intracellular glutathione and CoA pools (Nichenametla et al., 2022; Varghese et al., 2025). As an indispensable cofactor, CoA sits at the crossroads of carboxylic acid and fatty acid metabolism, influencing the activation and trafficking of both short- and long-chain acyl moieties (Leonardi et al., 2005; Foster, 2012). Crucially, the vast majority of intermediate metabolites participating in these two convergent pathways were consistently overrepresented in the DHC cohort. To transition from simple correlation to causal inference within this observational setup, we applied statistical mediation modeling (Rijnhart et al., 2021). A similar integrative paradigm was recently harnessed by Zhou et al. to map the serum-mediated pathways through which the microbiome interacts with age-related muscle attrition (Zhou et al., 2025). Paralleling their findings, our mediation framework crystallized Pyruvic acid, S-Adenosylmethionine, and Spermine as key candidate molecular nodes bridging the microbial footprint onto muscle FA deposition. Collectively, these systems-biology insights provide strong evidence that the *Agathobaculum*-*Collinsella*-*Enorma* consortium engages in cross-talk with distant skeletal tissues through a structured “gut microbiome-metabolites-muscle” axis. It is imperative to note, however, that while these multi-omic signatures offer a highly plausible mechanism, they do not inherently establish an absolute direct causality. Future translational investigations, particularly encompassing fecal microbiota transplantation (FMT) and germ-free validation models, remain indispensable to conclusively deconstruct the direct mechanical links governing this distal axis.

## 5 Conclusion

In conclusion, this study systematically reveals the “gut microbiota-metabolite-muscle” axis underlying variations in muscle FA deposition between DHC and LNC. Our findings demonstrate that DHC exhibited significantly higher accumulation of functional PUFAs (C20:5n3 and C22:5n3) and flavor-precursor MUFAs (C18:1n9c) in leg muscle compared to LNC. By integrating metagenomics, metabolomics, and mediation modeling, we identified a functional candidate axis: the dominant cecal *Agathobaculum*-*Collinsella*-*Enorma* consortium is closely associated with muscle FA accretion, a process bridged by key intermediate metabolites including Pyruvic Acid, S-Adenosylmethionine and Spermine enriched in “cysteine and methionine metabolism” and “pantothenate and CoA biosynthesis” pathways. This study clarifies a specific microbial consortium and intermediate metabolites that act as systemic regulators of muscle lipid composition. These findings enhance the understanding of the gut-muscle axis in poultry biology and offer microbial markers and metabolic targets for precision nutrition and molecular breeding strategies aimed at improving poultry meat quality.

## Data Availability

The raw sequence data reported in this paper have been deposited in the China National Center for Bioinformation (GSA: PRJCA070629) that are publicly accessible at https://www.cncb.ac.cn/.
