# Supplementary material for: Multi-omics reveals the associations between cecal candidate bacteria and muscle fatty acid composition variations in Daheng and Luning chickens
Source: Front Microbiol. 2026 Aug 31;17:1907226. doi: 10.3389/fmicb.2026.1907226 (PMC13572627; doi:10.3389/fmicb.2026.1907226)
Supplement: Supplementary file 1 [file Supplementary_file_2.pdf]

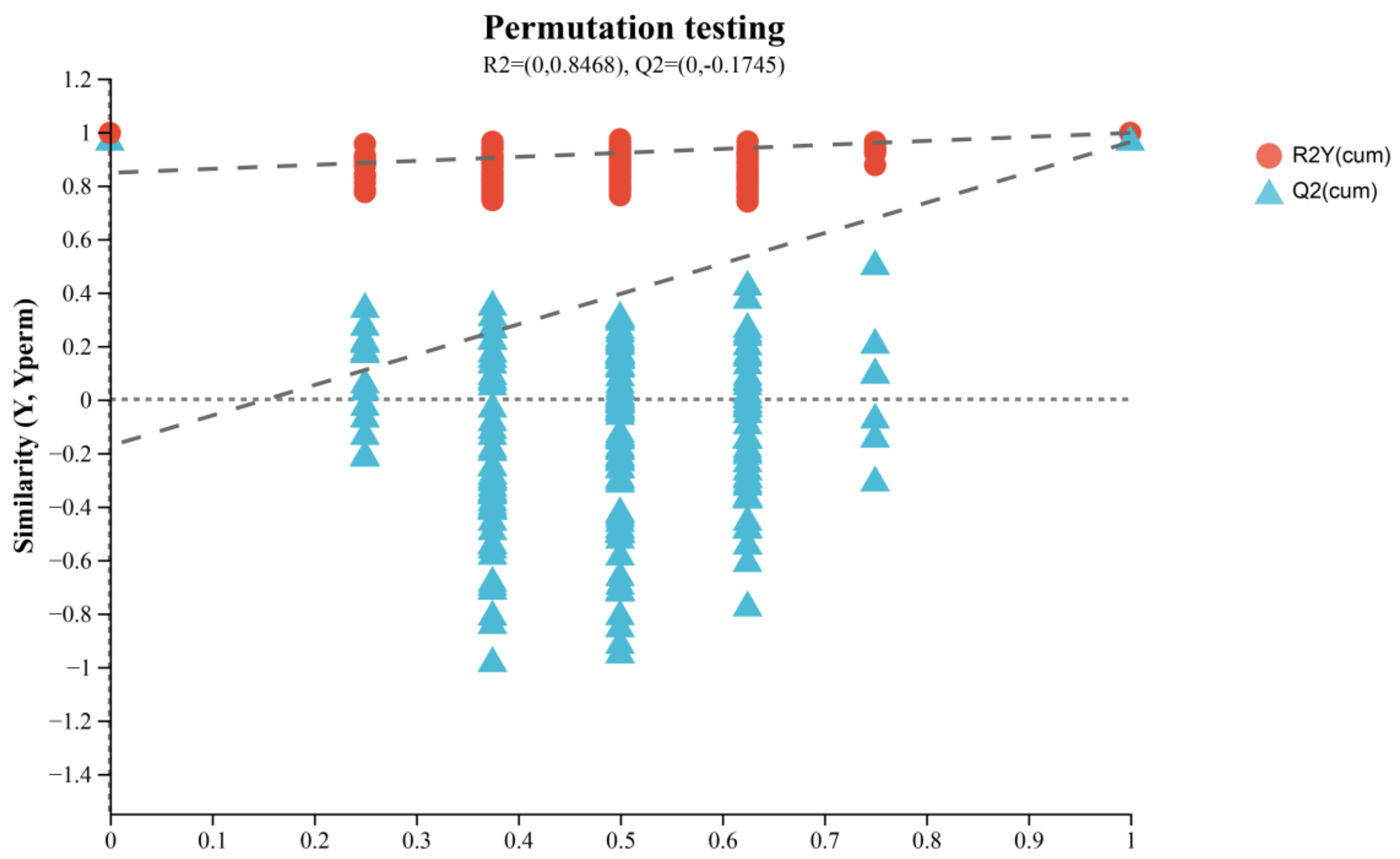

Figure S1 Permutation test for the OPLS-DA model of cecal content.

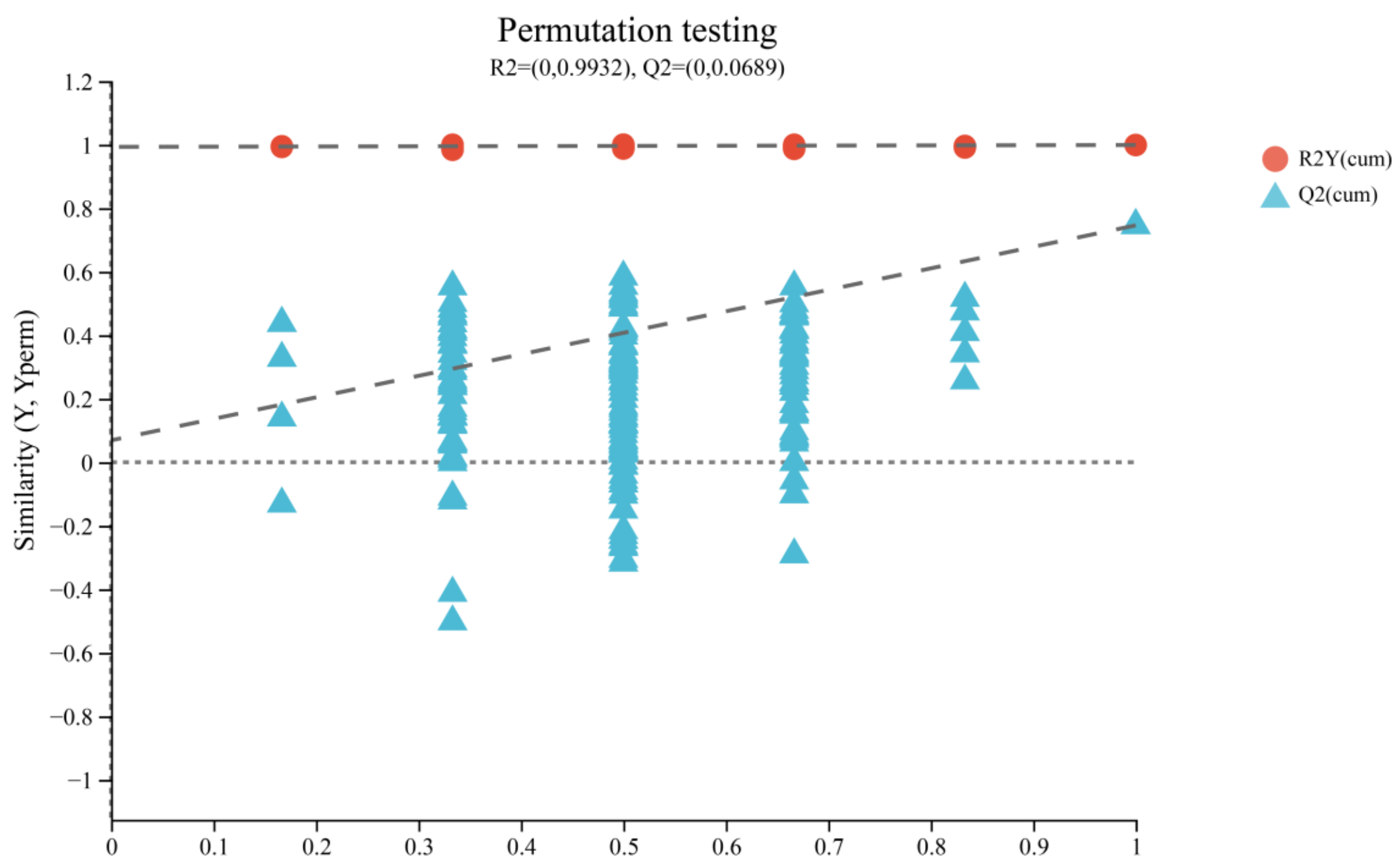

Figure S2 Permutation test for the OPLS-DA model of serum.

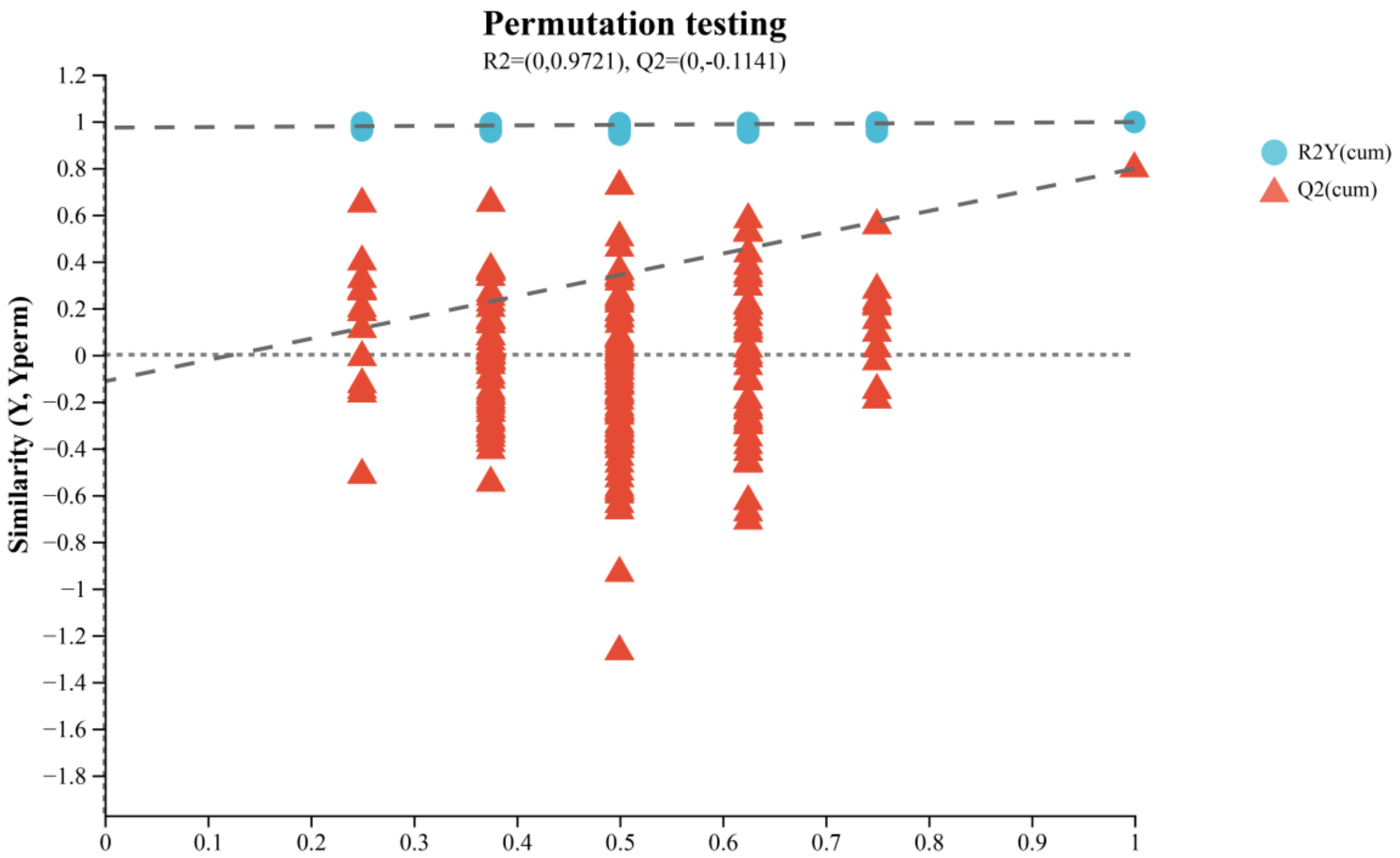

Figure S3 Permutation test for the OPLS-DA model of leg muscle.
