# Supplementary material for: Multi-omics reveals the associations between cecal candidate bacteria and muscle fatty acid composition variations in Daheng and Luning chickens
Source: Front Microbiol. 2026 Aug 31;17:1907226. doi: 10.3389/fmicb.2026.1907226 (PMC13572627; doi:10.3389/fmicb.2026.1907226)
Supplement: Supplementary file 2 [file Supplementary_file_1.docx]

**Supplementary file 1: The R scripts of Mantel test**

\# 加载必要的包

library(dplyr)

library(ggcor)

library(ggplot2)

\# 加载数据

micro <- read.table("micro100.txt", sep = "\t", header = TRUE)

metab <- read.table("metabolites.txt", sep = "\t", header = TRUE)

\# Mantel 检验分组（使用列名，避免索引错误）

spec_groups <- list(

Spec01 = colnames(metab)[1:1335],

Spec02 = colnames(metab)[1336:1405],

Spec03 = colnames(metab)[1406:1461]

)

\# 执行 Mantel 检验并添加分组标签

mantel <- mantel_test(metab, micro, spec.select = spec_groups, permutations = 999) %>%

mutate(rd = cut(r, breaks = c(-Inf, 0.2, 0.4, Inf),

labels = c("< 0.2", "0.2 - 0.4", ">= 0.4")),

pd = cut(p.value, breaks = c(-Inf, 0.01, 0.05, Inf),

labels = c("< 0.01", "0.01 - 0.05", ">= 0.05")))

\# 绘图

quickcor(micro, type = "upper") +

geom_circle2() + scale_fill_gradient2(low = "#0033FF", mid = "white", high = "#FF3300") +

anno_link(aes(colour = pd, size = rd), data = mantel) +

scale_size_manual(values = c(0.5, 1, 2)) +

scale_colour_manual(values = c("#D95F02", "#1B9E77", "#A2A2A288")) +

guides(size = guide_legend(title = "Mantel's r",

override.aes = list(colour = "grey35"),

order = 2),

colour = guide_legend(title = "Mantel's p",

override.aes = list(size = 3),

order = 1),

fill = guide_colorbar(title = "Pearson's r", order = 3))

**Supplementary file 2: The R scripts of Mediation analysis**

\# 加载必需包

library(dplyr)

library(mediation)

\# 1. 读取3个txt数据文件（制表符分隔，匹配你的数据格式）

\# 微生物数据：microbiota.txt → 命名为micro

micro <- read.delim("microbiota.txt", sep = "\t", header = TRUE,

check.names = FALSE, stringsAsFactors = FALSE)

\# 代谢物数据：metabolite.txt → 命名为metab

metab <- read.delim("metabolite.txt", sep = "\t", header = TRUE,

check.names = FALSE, stringsAsFactors = FALSE)

\# 脂肪酸数据：FAs.txt → 命名为fa

fa <- read.delim("FAs.txt", sep = "\t", header = TRUE,

check.names = FALSE, stringsAsFactors = FALSE)

\# 2. 数据合并+自动创建分组变量Group（关键！DH=实验组/LN=对照组）

\# 按SampleID内连接3张表，仅保留共同样本

data <- micro %>%

inner_join(metab, by = "SampleID") %>%

inner_join(fa, by = "SampleID")

\# 从SampleID提取分组：以DH开头=Treatment(实验组)，LN开头=Control(对照组)

data$Group <- ifelse(substr(data$SampleID, 1, 2) == "DH", "Treatment", "Control")

\# 分组转为因子（统计模型必备）

data$Group <- factor(data$Group, levels = c("Control", "Treatment"))

\# 查看数据基本信息（验证是否正确）

cat("数据合并后样本量：", nrow(data), "\n")

cat("分组情况：\n")

print(table(data$Group))

cat("前5行数据预览：\n")

print(head(data[, c("SampleID", "Group")]))

\# 3. 数据标准化（仅标准化数值列，跳过SampleID和Group，避免分组被标准化）

data_scaled <- data

\# 确定需要排除的列：SampleID(第1列) + Group列

exclude_cols <- c(1, which(colnames(data_scaled) == "Group"))

\# 提取数值列并标准化

sub_data <- data_scaled[, -exclude_cols, drop = FALSE]

\# 转为数值型（避免字符型干扰）

sub_data <- data.frame(lapply(sub_data, function(x) suppressWarnings(as.numeric(x))))

\# Z-score标准化（均值0，标准差1）

sub_data <- as.data.frame(scale(sub_data))

\# 替换回原数据框

data_scaled[, -exclude_cols] <- sub_data

\# 4. 提取三类核心变量名（自动匹配文件列名，无需手动修改）

microbes <- colnames(micro)[-1]	# 微生物变量（排除SampleID）

metabolites <- colnames(metab)[-1]	# 代谢物变量（排除SampleID）

phenotypes <- colnames(fa)[-1]	# 脂肪酸(FAs)变量（排除SampleID）

cat("微生物数量：", length(microbes), "\n")

cat("代谢物数量：", length(metabolites), "\n")

cat("脂肪酸数量：", length(phenotypes), "\n")

\# 5. 初始化结果保存对象

results_list <- list()

count <- 1

\# 6. 三重循环批量中介分析（X=微生物→M=代谢物→Y=脂肪酸，带分组校正）

\# 关键优化：分组校正+16样本稳健标准误+错误捕获（避免单个模型报错中断整体运行）

for (x in microbes) { # 遍历所有微生物

for (m in metabolites) { # 遍历所有代谢物

for (y in phenotypes) { # 遍历所有脂肪酸

\# 构建带分组校正的模型公式（核心：+ Group）

\# 模型M：代谢物 ~ 微生物 + 分组（路径a）

formula_M <- as.formula(paste0("`", m, "` ~ `", x, "` + Group"))

\# 模型Y：脂肪酸 ~ 微生物 + 代谢物 + 分组（路径b+c'）

formula_Y <- as.formula(paste0("`", y, "` ~ `", x, "` + `", m, "` + Group"))

\# 拟合线性模型，捕获错误（如完全共线性）

model.M <- try(lm(formula_M, data = data_scaled), silent = TRUE)

model.Y <- try(lm(formula_Y, data = data_scaled), silent = TRUE)

\# 跳过拟合失败的模型

if (inherits(model.M, "try-error") || inherits(model.Y, "try-error")) next

\# 执行中介分析（Bootstrap法+16样本稳健标准误robustSE=TRUE）

med <- try(

mediate(

model.m = model.M,

model.y = model.Y,

treat = x, # 自变量：微生物

mediator = m, # 中介变量：代谢物

boot = TRUE, # 开启Bootstrap抽样（更稳健的置信区间）

sims = 2000, # Bootstrap抽样次数（2000次）

robustSE = TRUE, # 小样本（16例）必备，稳健标准误

conf.level = 0.95	# 95%置信区间

),

silent = TRUE

)

\# 跳过中介分析失败的组合

if (inherits(med, "try-error")) next

\# 提取中介分析核心结果

res <- try(

data.frame(

Microbe = x,

Metabolite = m,

Phenotype = y,

\# 路径a：微生物→代谢物（系数+P值）

a_coef = coef(summary(model.M))[2, 1],

a_p	= coef(summary(model.M))[2, 4],

\# 路径b：代谢物→脂肪酸（控制微生物+分组，系数+P值）

b_coef = coef(summary(model.Y))[3, 1],

b_p	= coef(summary(model.Y))[3, 4],

\# 间接效应（ACME：中介效应，核心关注）

ACME = med$d0,

ACME_LCI = med$d0.ci[1],

ACME_UCI = med$d0.ci[2],

ACME_P = med$d0.p,

\# 直接效应（ADE：微生物直接→脂肪酸）

ADE = med$z0,

ADE_LCI = med$z0.ci[1],

ADE_UCI = med$z0.ci[2],

ADE_P = med$z0.p,

\# 总效应（直接+间接）

Total_Effect = med$tau.coef,

Total_Effect_LCI = med$tau.ci[1],

Total_Effect_UCI = med$tau.ci[2],

Total_Effect_P = med$tau.p,

\# 中介效应占总效应的比例

Prop_Mediated = med$n0,

Prop_Mediated_LCI = med$n0.ci[1],

Prop_Mediated_UCI = med$n0.ci[2],

Prop_Mediated_P = med$n0.p,

stringsAsFactors = FALSE

),

silent = TRUE

)

\# 跳过结果提取失败的组合

if (inherits(res, "try-error")) next

\# 保存结果到列表

results_list[[count]] <- res

count <- count + 1

}

}

}

\# 7. 结果整合+BH多重校正（避免假阳性，论文必备）

\# 将列表转为数据框

results_df <- do.call(rbind, results_list)

\# 对核心P值做BH多重校正（FDR）

results_df$ACME_P_BH <- p.adjust(results_df$ACME_P, method = "BH")	# 中介效应P值校正

results_df$ADE_P_BH <- p.adjust(results_df$ADE_P, method = "BH")	# 直接效应P值校正

results_df$Total_Effect_P_BH <- p.adjust(results_df$Total_Effect_P, method = "BH") # 总效应P值校正

\# 8. 筛选显著结果+导出文件（3个结果表，满足不同需求）

\# 筛选：中介效应原始P<0.05

results_p_sig <- results_df %>% filter(ACME_P < 0.05)

\# 筛选：中介效应BH校正后P<0.05（最严格）

results_fdr_sig <- results_df %>% filter(ACME_P_BH < 0.05)

**Supplementary file 3: The Mediation analysis models and key parameters**

To determine whether intermediate cecal/serum metabolites statistically mediated the linkage between specific gut microbial taxa and muscle fatty acid deposition, causal mediation analysis was conducted within a counterfactual framework using the mediation package (v4.5.0) in R.

For each candidate axis, a linear mediator model and a linear outcome model were fitted as follows:

Mediator Model:

$$M_{i}=\alpha_{0}+\alpha_{1}X_{i}+\tau_{1}C_{i}+\varepsilon_{1i}$$

Outcome Model:

$$Y_{i}=\gamma_{0}+\gamma^{'}X_{i}+\beta M_{i}+\tau_{2}C_{i}+\varepsilon_{2i}$$

Where:

$X_{i}$ represents the independent variable (relative abundance of core bacterial genera);

$M_{i}$ represents the potential mediator node (cecal/serum metabolites)

$Y_{i}$ represents the outcome variable (specific muscle fatty acid traits)

$C_{i}$ denotes potential confounding covariates

$\alpha_{1}$, $\beta$, and $\gamma^{'}$ are regression coefficients, and $\varepsilon_{1i}$, $\varepsilon_{2i}$ are random error terms.

The **Average Causal Mediation Effect (ACME)**, representing the indirect effect transmitted through the metabolite ***M***, was calculated as the product of coefficients (ACME = $\alpha_{1}$ × $\beta$). The **Average Direct Effect (ADE)** was denoted by $\gamma^{'}$, and the Total Effect (TE) was calculated as TE = ADE +ACME.

The **Proportion Mediated** was derived as:

$$Proportion Mediated=\frac{ACME}{TE}\times100\%$$

Statistical significance and 95% confidence intervals (CIs) for ACME, ADE, and Proportion Mediated were estimated via non-parametric bootstrapping with **2,000 iterations** (quasi-Bayesian approximation). Candidate pathways satisfying both *P*_ACME_ < 0.05, and *P*_Prop_ < 0.05 were recognized as statistically significant mediation axes.
